# Rapid guidelines – timely and important guidance needed for setting standards and best practices

**DOI:** 10.1186/s12961-018-0328-7

**Published:** 2018-06-12

**Authors:** Tikki Pang, Gianna Gayle Herrera Amul

**Affiliations:** Lee Kuan Yew School of Public Policy, National University of Singapore, Singapore, Singapore

In an increasingly unstable world faced with multiple health threats and emergencies, practitioners and decision-makers are increasingly in need of evidence-based rapid guidelines (RGs) in order to guide the implementation of public health measures to mitigate the negative effects of such threats. The outbreak of Ebola in West Africa in 2015 and a potential Ebola outbreak currently occurring in the Democratic Republic of Congo, together with the current, looming threat of avian influenza H3N2 and H7N9 in Asia, are just some examples of the explosive and rapidly developing infectious disease outbreaks which could benefit from the availability of such guidelines, for example, in the areas of detection, containment, treatment, public health measures and, importantly, risk communication. The need is especially great in fragile states in the developing world, which have limited health facilities, infrastructure, health workers and resources, as illustrated by the recent outbreak of cholera in Yemen killing nearly 2000 people. In countries with weakened health systems, limited resources and competing priorities, proper and evidence-based guidance during health emergencies becomes even more important for optimal decision-making. In addition, public health emergencies caused by natural disasters (e.g. earthquakes, tsunamis), extreme weather events (e.g. earthquakes, floods, landslides, heatwaves) and the deliberate or accidental release of biological and chemical agents (e.g. the recent use of VX nerve gas in an assassination in Malaysia), pose an exceptional but urgent need for the timely availability of RGs to prevent a rapid deterioration of any public health emergency.

In practical terms, RGs are arguably most helpful for national and local governments in guiding their responses to public health crises and emergencies. However, and at the same time, in major, large-scale emergencies where external actors are involved in an international response (e.g. the Ebola outbreak in West Africa and the Southeast Asia tsunami), such RGs are also invaluable to humanitarian organisations (e.g. MSF), non-governmental organisations and other front-line responders (e.g. the military/armed forces from assisting countries) which often work hand-in-hand with national teams in mitigating the effects of such crises.

RGs have been developed, for example, for the treatment of cancer [1] and tuberculosis [2] and for public health emergencies [3]. Several initiatives have also focused on rapid evidence assessments, including the Cochrane Collaboration’s Rapid Review Methods Group [4]. While some attempts have been previously made to provide guidance for the rapid assessment of evidence [5, 6], there has not been a more general, thorough and systematic study of the current practices and methods in the development of RGs. The value of research evidence has previously been highlighted in the 2006 *Health Research Policy and Systems* series, in collaboration with the WHO Advisory Committee on Health Research’s Subcommittee on the Use of Research Evidence on ‘Improving the use of research evidence in guideline development’, which was rooted in the need of WHO to maximise research evidence for its recommendations, guidelines and policies [7].

In this regard, the ‘Development of rapid guidelines’ series recently published in *Health Research Policy and Systems* represent significant advances in this important field. The first paper by Kowalski et al. [8] is a rigorous and comprehensive systematic review of current practices and methods in the development of RGs. Analysing 35 RGs in diverse fields developed by various organisations, they show that the rationale and reasons for developing RGs were related mainly to health emergencies, rapid increases in cases of a particular condition, unusually severe disease, or the emergence of new evidence in relation to treatment modalities. They conclude, however, that there is a lack of standardised nomenclature and definitions regarding RGs and inconsistencies in the methods presented in manuals and in the guidelines that are produced.

Taking the above into account, and using a ‘case study’ approach, the second paper in the series, by Flórez et al. [9], studied RG development at WHO through a series of interviews with rapid guideline developers among the staff. While their study underscored the important finding of the first paper on the importance and rationale for RG development, and obtained valuable insights into RG development, they conclude that much more research needs to be done regarding standardisation of processes relating to critical timelines, panel composition, the peer-review process, the conduct of meetings and the sources of evidence.

Building on these findings, the third paper, by Morgan et al. [10], defined a set of principles to guide the development of RGs. They identified 21 guiding principles which they propose should guide the planning and development of RGs while also maintaining the rigor of a standardised and transparent process. The principles span a range of key dimensions of RG development, including organisation and planning, priority-setting, group membership and processes, target audience, conflicts of interest, question generation, outcome prioritisation processes, evidence source and quality, developing recommendations and assessing the strength, implementation, feasibility and equity of recommendations, reporting and peer review, dissemination and implementation, and evaluation and updating of RGs. The guiding principles serve as a standardised model for developing and assessing RGs for public health emergencies not only for guideline developers but also for RG users and policy-makers. The principles are rigorous but flexible enough to be applied in critical situations that require urgent evidence-based guidance.

It is hoped that the publication of these important studies will be a stepping stone towards the development and setting of a much needed set of robust norms and standards and best practices for RG development, perhaps spearheaded by WHO and a revival and strengthening of its Guidelines Review Committee, in close collaboration with the Cochrane Collaboration, academia and other relevant organisations [11]. The need and the importance of RGs will continue to grow in the future and such a move is timely, important and necessary.
